# Supplementary material for: CED-3 caspase acts with miRNAs to regulate non-apoptotic gene expression dynamics for robust development in C. elegans
Source: eLife. 2014 Dec 30;3:e04265. doi: 10.7554/eLife.04265 (PMC4279084; doi:10.7554/eLife.04265)
Supplement: Supplementary file 1. — Definition of phenotypes scored in this study. DOI: http://dx.doi.org/10.7554/eLife.04265.037 [file elife04265s008.docx]

**Supplemental Table 1: Definition of phenotypes scored in this study.**

| **Phenotype** | **Description** | **Definition** |
| --- | --- | --- |
| Normal | Superficially wild type | Similar to control strain phenotype on mock RNAi |
| No interaction | No enhancer phenotype | Similar to control strain on the given RNAi |
| Rup | Ruptured through vulva | >50% F0 animals had ruptured through the vulva |
| Bmd | Body morphology defect | >50% of F0 population dysmorphic and/or shriveled |
| Egl | Egg laying defect | >50% of F0 population severe Egl (usually no eggs were layed) |
| Bag | Bag of worms | >50% F0 animals had bagged (grouped with Egl) |
| F0-Slu | F0 Sluggish | >50% F0 worms with obviously reduced mobility |
| Lva | Larval arrest | >50% F0 animals arrested before reaching adulthood |
| Emb | Embryonic lethal | F1 Eggs with no or very few hatchlings (accumulation of eggs in well) |
| Red | Reduced brood size | >90% reduction in brood size (essentially <1 offspring per adult) |
| Bsv | Brood size variant | 50%-90% reduction in brood size |
| F1-Prl | F1 Paralyzed | >50% F1 worms did not move at all, maybe dead or very severe unc |
| F1-Slu | F1 Sluggish | >50% F1 worms with obviously reduced mobility |
