# Supplementary material for: CED-3 caspase acts with miRNAs to regulate non-apoptotic gene expression dynamics for robust development in C. elegans
Source: eLife. 2014 Dec 30;3:e04265. doi: 10.7554/eLife.04265 (PMC4279084; doi:10.7554/eLife.04265)
Supplement: Supplementary file 2. — List of ain-1 and ain-2 genetic interactors identified in this study and their relevant phenotypes in brief. RNAi clones are listed alphabetically by gene name. Relevant phenotypes indicated for the given strains are defined in Supplementary file 1. DOI: http://dx.doi.org/10.7554/eLife.04265.038 [file elife04265s009.docx]

**Supplemental Table 2: List of *ain-1* and *ain-2* genetic interactors identified in this study and their relevant phenotypes in brief.** RNAi clones are listed alphabetically by gene name. Relevant phenotypes indicated for the given strains are defined in supplemental Table 1.

| **RNAi** | **Phenotype** | | |
| --- | --- | --- | --- |
| ***Gene*** | ***ain-1;rrf-3*** | ***ain-2;rrf-3*** | ***rrf-3*** |
| *abu-1* | Normal | Bsv | Normal |
| *asb-2* | Bsv | Red, F1-Slu | Bsv |
| *B0238.12* | Rup, Bsv | Bsv | Normal |
| *B0281.5* | Rup, Egl | Normal | Normal |
| *bath-43* | Bmd, Bsv | Normal | Normal |
| *C04F5.8* | F0-Slu, Bsv | Bsv | Normal |
| *C06G1.5* | Egl | Red | Bsv |
| *C06G3.8* | Bsv, F0-Slu | Normal | Normal |
| *C08H9.2* | Red, F1-Slu | Red, F1-Slu | Bsv |
| *C24B5.4* | Bsv, F0-Slu | Red | Normal |
| *C25G4.6* | Red | Bsv | Normal |
| *C44B7.2* | F0-Slu, Bsv | Bsv | Normal |
| *C45G9.12* | Rup, F0-Slu, Bsv | Normal | Normal |
| *C50F4.4* | Rup, Egl | Bsv | Normal |
| *calu-1* | Bsv | Bmd, Red | Bsv |
| *capg-1* | Egl, F0-Slu | Emb | Normal |
| *cdc-48.3* | Bmd, Bsv | Bsv | Normal |
| *cdt-2** | Rup, Bmd, Egl, F1-Slu | Bsv | Bsv |
| *ced-3* | Emb | Bsv | Bsv |
| *ceh-18** | F0-Slu, Bmd, Bsv | Red | Bsv |
| *ceh-38* | Bsv, F0-Slu, F1-Slu | F0-Slu | Normal |
| *chk-2* | Emb | Emb | Red |
| *col-144* | F0-Slu, Bsv | Normal | Normal |
| *csn-1* | Egl | Red | Bsv |
| *csn-4* | Bmd, Red | Bmd, Emb | Bsv |
| *csn-5* | Bmd, Red | Red | Bsv |
| *cyn-3* | Bmd, Bsv | Bsv | Bsv |
| *cyp-42A1* | F0-Slu | Bsv | Normal |
| *daf-12** | Rup, Egl, F0-Slu | Bsv | Normal |
| *dcn-1* | Red | Bsv | Bsv |
| *djr-1.1* | Egl, Rup, Bsv | Normal | Normal |
| *dpy-11* | F1-Prl | F1-Prl, Bsv | Bsv |
| *dpy-2* | Red | Red | Bsv |
| *dpy-21** | Egl, Bmd, F0-Slu | Bsv | Normal |
| *eel-1* | Bmd, Bsv | F0-Slu, F1-Slu, Bsv | Bsv |
| *eff-1* | Bmd, F0-Slu | F0-Slu | Normal |
| *F14D7.3* | Normal | Bsv | Normal |
| *F16B12.6* | Red | Red | Bsv |
| *F21D5.1* | Emb | Emb | Red |
| *F23C8.6* | Egl, F0-Slu | Red, F0-Slu | Bsv |
| *F43D2.3* | F0-Slu, Bsv | Bsv | Normal |
| *F52C6.12* | Bmd, F0-Slu, Bsv | Bsv, F0-Slu | Bsv |
| *F52C6.3* | Egl, Bmd, F0-Slu | Red, F0-Slu | Bsv |
| *F53C3.4* | Bsv | Emb | Normal |
| *fat-3* | Egl, F2-Slu | Red | Bsv |
| *flp-11* | Bsv | Bsv | Normal |
| *gcy-11* | Rup, Bmd, Red | Bsv | Bsv |
| *glh-1* | Bmd, Bsv | Red | Bsv |
| *grl-31* | Egl, F0-Slu,Bmd | Egl | Red |
| *H06I04.1* | Bmd, Bsv | Normal | Normal |
| *his-24* | Bsv, F0-Slu | Red | Normal |
| *hmg-12* | F0-Slu, Bsv | Normal | Normal |
| *hpo-18* | Bsv | Red | Bsv |
| *hrp-1* | Rup, Egl | Red | Bsv |
| *inos-1* | Rup, F0-Slu, Red | Red, F0-Slu | Bsv, F1-Unc |
| *ins-26* | BSV, Rup | Red | Normal |
| *K01C8.1* | Rup, Bmd, F0-Slu, Egl | Egl | Egl |
| *K03B4.2* | F0-Slu, Bsv | Bsv | Normal |
| *K07E12.2* | Rup, Bmd, F0-Slu, Bsv | Normal | Normal |
| *lag-2** | Rup, Bmd, Bsv | Red | Bsv |
| *lfe-2* | Bsv | Red | Normal |
| *lin-46* | Rup, F0-Slu, Bsv | Bsv | Bsv |
| *lsl-1* | Red | Red | Bsv |
| *lsm-4* | Egl, Unc | Red | Bsv |
| *lst-3** | Normal | Bsv | Normal |
| *mev-1* | Rup,Red | Bsv | Normal |
| *mig-38* | Bmd, Red, F0-Slu | Bsv, F1-Slu | Bsv |
| *mrpl-11* | Bsv, F0-Slu | Red | Bsv |
| *mrpl-2* | Egl | Red | Red |
| *mrpl-47* | Egl | Red | Bsv |
| *mrps-17* | Bmd, Red, F0-Slu | Bsv | Bsv |
| *msi-1* | Rup, Bmd, Red | Bsv | Normal |
| *nfm-1* | Bmd, Bsv, F0-Slu | Bsv | Normal |
| *nhr-60* | F0-Slu, Bsv | Normal | Normal |
| *pcaf-1* |  | Red, F0-Slu, F1-Slu | Bsv |
| *pfd-2* | Red | Bsv, F1-Prl, F0-Slu | Bsv |
| *pfd-3* | Egl, F0-Slu | Egl | F0-Slu, Bsv |
| *pqn-85* | Red, F0-Slu | Bsv | Bsv |
| *ptr-24* | Bmd, Egl, F0-Slu | Bsv | Bsv |
| *pup-2* | Egl, Bmd, F0-Slu | Emb | Bsv |
| *R11H6.2* | Bmd, Bsv | Bsv | Normal |
| *rack-1* | Egl | Red | Bsv |
| *rad-51* | Egl, Emb | Egl, Emb, F0-Slu | Bsv |
| *ral-1* | Red, F0-Slu | Red | Bsv |
| *rap-1* | Rup. Egl, Bsv | Normal | Normal |
| *rfp-1* | Bmd, Bsv | Red | Bsv |
| *sars-2* | Egl, Red | Egl, Red | Bsv |
| *scl-12* | Normal | Red | Normal |
| *sex-1** | Emb,F1-Prl | Emb | Bsv |
| *skr-19* | Bmd, Egl, F0-Slu, Red | Red | Bsv |
| *skr-7* | Bsv, F0-Slu | Normal | Normal |
| *smk-1* | Emb | Emb | Bsv |
| *sod-2* | Rup, Bsv | Bsv | Bsv |
| *sod-3* | Bsv | Rup, Bsv | Normal |
| *spe-19* | F0-Slu, Bsv | Bsv | Normal |
| *sss-2* | F0-Slu, Bsv | Bsv | Normal |
| *str-232* | Rup, F0-Slu, Bsv | Normal | Normal |
| *T05B11.1* | Rup, Bmd, F0-Slu, Bsv, F1-Slu | F0-Slu, Bsv | Normal |
| *T12A2.7* | Red | Egl | Bsv |
| *T16H12.1* | Bmd, Red | Red | Bsv |
| *T25D3.4* | F0-Slu, Bsv | Bsv | Bsv |
| *T26A5.4* | Egl, F0-Slu | Red, F1-Slu | Bsv |
| *T27E9.1* | Red | Red | Bsv |
| *tbp-1* | Rup, Bsv | Bsv | Bsv |
| *tpst-1* | Egl, Red | L4 Arrest | Bsv |
| *tsp-17* | Bmd, Egl, F0-Slu | Egl | Egl |
| *unc-39* | Normal | F1-Slu, Bsv | Normal |
| *unc-94* | Red, F0-Slu | Egl | Bsv |
| *vap-1* | Egl, F0-Slu | Normal | Normal |
| *W01A8.5* | Bsv, F0-Slu | Normal | Normal |
| *W02H5.8* | Rup, Egl, Bmd, F0-Slu | Egl | Bsv |
| *W03F9.3* | Bmd, F0-Slu, Bsv | Normal | Normal |
| *W06D12.6* | Rup, Egl, Bmd | Bsv, F1-Prl | Normal |
| *W07G4.3* | Rup, Bmd, Bsv, F1-Slu | Normal | Normal |
| *wrt-1* | Bsv, Bmd, F0-Slu | Red | Bsv |
| *Y102E9.3* | Rup, Bmd, Red, F0-Slu | Bmd, Red, F0-Slu | Bsv |
| *Y17D7B.4* | Rup, Red, F0-Slu | Bsv | Bsv |
| *Y39C12A.1** | Bmd, Red | Bmd, Egl | Bsv |
| *Y54G2A.26* | Bsv, F0-Slu | Bsv | Bsv |
| *Y59A8B.10* | Bmd, F0-Slu | Bsv | Normal |
| *Y95B8A.6* | Bmd, F0-Slu, Bsv | Bsv | Bsv |
| *ZC395.10* | Red, F0-Slu | Bsv | Bsv |
| *ZK381.2* | Rup, Bmd, Bsv | Bsv | Bsv |
| *ZK484.1* | Bmd, Bsv | Bsv | Normal |
| *ZK652.8* | Rup, Bmd | Bsv | Normal |
| *ztf-8* | Bsv | Bsv | Normal |

*These genes have previously been shown to interact with the miRNA pathway.
