# Supplementary material for: CED-3 caspase acts with miRNAs to regulate non-apoptotic gene expression dynamics for robust development in C. elegans
Source: eLife. 2014 Dec 30;3:e04265. doi: 10.7554/eLife.04265 (PMC4279084; doi:10.7554/eLife.04265)
Supplement: Supplementary file 3. — Phenotypes observed for reverse confirmation test with ain-1 and ain-2 RNAi. Effects indicated are relative to the given mutant strain phenotype on mock RNAi. These are results for one generation on the indicated RNAi and not with RNAi enhancing mutations. DOI: http://dx.doi.org/10.7554/eLife.04265.039 [file elife04265s010.docx]

**Supplemental Table 3: Phenotypes observed for reverse confirmation test with *ain-1* and *ain-2* RNAi.** Effects indicated are relative to the given mutant strain phenotype on mock RNAi. These are results for one generation on the indicated RNAi and not with RNAi enhancing mutations.

| ***Gene(allele)*** | ***ain-1* Enhancer** | ***ain-1* RNAi**  **Phenotype** | ***ain-2* Enhancer** | ***ain-2* RNAi**  **Phenotype** |
| --- | --- | --- | --- | --- |
| *ceh-18(mg57)* | Strong | F0 all dead; F1 Bsv | Mild | F1 Bsv |
| *djr-1.1 (tm0918)* | Strong | F0 >50% dead; F1 10-20% Bsv | Mild | F1 Bsv |
| *msi-1 (os1)* | Strong | F0 >70% Rup, 100% Slu; F1: 100% Pvl, Bsv | Mild | F0 few Rup; F1 Bsv |
| *unc-94(su177)* | Strong | F0 >50% Rup; F1 Bsv, 20% Pvl, | Mild | F0 A few Rup; F1 Bsv |
| *dpy-21(e428)* | Strong | F0 all dead; F1 Red | Moderate | F0 10-50% dead |
| *mev-1(kn1)* | Strong | F0 >90% dead; F1 Red | Moderate | F0 20% dead |
| *ced-3 (n1286)* | Strong | Delay in development; F1 Bsv, Emb | No | Normal to Few offspring (Highly variable) |
| *ceh-18(ok1082)* | Strong | F0 all dead; F1 delay in development | No | Normal |
| *daf-12(rh286)* | Strong | F0 >60% dead; F1 Bsv | No | Normal |
| *glh-1(ok439)* | Strong | F0 >50% Rup | No | Normal |
| *sod-3(tm760)* | Strong | Strong delay in development; F1 Bsv | No | Normal |
| *skr-7(tm3532)* | Strong | Strong delay in development; F1 Bsv | Strong | F1 Red |
| *C06G1.5 (tm5279)* | Moderate | 10% Rup; F1 Bsv | Mild | F1 Bsv |
| *tsp-17(tm5169)* | Moderate | F1 Bsv | Mild | F1 Bsv |
| *chk-2(gk212)* | Moderate | F0 dead; F1 Bsv | No | F1 Bsv |
| *rap-1 (pk2082)* | Moderate | F0 Enhanced lethality, delay in development; F1 Bsv | No | No interaction |
| *F16B12.6 (gk1118)* | Moderate | All F0 dead; F1 Red | Mild | F0 30% Rup; F1 Bsv |
| *bath-43 (tm770)* | Moderate | F0 30-40% dead, Rup; F1 Bsv, delay in development | No | No interaction |
| *F53C3.4 (ok2748)* | Moderate | F0 50% dead/dying; F1 Red | Mild | F0 10% dead |
| *ced-3 (n717)* | Mild | F1 Bsv | No | Normal |
| *cyn-3(tm2573)* | Mild | F0 10% Rup; F1 Bsv | No | Normal |
| *glh-1(gk100)* | Mild | F0 20%-30% Rup | No | Normal |
| *W02H5.8 (tm5127)* | Mild | F0 20%-30% Rup | No | Normal |
| *C25G4.6 (tm3228)* | Mild | F1 Bsv, delay in development | No | Normal |
| *pup-2(tm4344)* | Mild | F0 20-40% Rup; F1 Bsv | No | F0 A few Bag; F1 Bsv |
| *sars-2 (tm3144)* | Mild | F0 30-50% Rup | No | No interaction |
| *nhr-60 (ok1622)* | Mild | F1 Bsv | No | No interaction |
| *ins-26 (tm1983)* | mild | F0 10-70% Rup | No | No interaction |
| *Y67D8C.5 (ok1575)* | No | No interaction | Moderate | F1 Bsv, reduced eggs |
| *Y95B8A.6 (tm5312)* | No | A few Rup | No | Normal |
| *vap-1(ok392)* | No | L3 stages under-represented | No | No interaction |
| *C08H9.2 (ok1071)* | No | A few Rup | No | No interaction |
