# Supplementary material for: CED-3 caspase acts with miRNAs to regulate non-apoptotic gene expression dynamics for robust development in C. elegans
Source: eLife. 2014 Dec 30;3:e04265. doi: 10.7554/eLife.04265 (PMC4279084; doi:10.7554/eLife.04265)
Supplement: Supplementary file 4. — List of C. elegans strains and relevant genotypes used in this study. DOI: http://dx.doi.org/10.7554/eLife.04265.040 [file elife04265s011.docx]

**Supplemental Table 4: List of *C. elegans* strains and relevant genotypes used in this study.**

| **Screen Study** | | ***ced-3* Study** |
| --- | --- | --- |
| *ain-1(ku322)* | | *ced-1(e1735)* |
| *ain-1(tm3681)* | | *ced-3(n1286)* |
| *ain-2(tm2432)* | | *ced-3(n717)* |
| *rrf-3(pk1426)* | | *ced-1(e1735);ced-3(n717)* |
| *ain-1(ku322);rrf-3(pk1426)* | | *ain-1(ku322);ced-3(n1286)* |
| *ain-2(tm2432);rrf-3(pk1426)* | | *ain-1(ku322);ced-3(n717)* |
| **Reverse Confirmation** | | *ain-1(tm3681);ced-3(n1286)* |
| *daf-12(rh286)* | | *ain-1(tm3681);ced-3(n717)* |
| *dpy-21(e428)* | | *ced-4(n1162)* |
| *ceh-18(mg57)* | | *ain-1(ku322);ced-4(n1162)* |
| *unc-94(su177)* | | *ain-1(ku322);ceh-18(mg57)* |
| *msi-1 (os1)* | | *miR-246(n4636);ced-3(n1286)* |
| *C08H9.2 (ok1071)* | | *miR-1(n4101);ced-3(n1286)* |
| *ceh-18(ok1082)* | | *miR-48(n4097);ced-3(n1286)* |
| *nhr-60 (ok1622)* | | *miR-84(n4037);ced-3(n1286)* |
| *F53C3.4(ok2748)* | | *mir-48(n4097);miR-84(n4037);ced-3(n1286)* |
| *glh-1(ok439)* | | *miR-1(n4101);miR-84(n4037);ced-3(n1286)* |
| *mev-1(kn1)* | | *nuc-1(e1392)* |
| *djr-1.1 (tm0918)* | | *ain-1(ku322);nuc-1(e1392)* |
| *ins-26 (tm1983)* | | *ced-3 (n2427);nls106* |
| *cyn-3(tm2573)* | | *mcd-1(n3376);ced-3 (n2427);nls106* |
| *sars-2 (tm3144)* | | *mcd-1(n3376)II;nls106* |
| *C25G4.6(tm3228)* | | *ced-3(n717);nls106* |
| *skr-7(tm3532)* | | *ain-1(ku322);ced-3 (n2427);nls106* |
| *pup-2(tm4344)* | | *unc-119(e2498::Tc1); wIs51* |
| *W02H5.8(tm5127)* | | *ain-1(ku322);SCM::gfp* |
| *tsp-17(tm5169)* | | *ain-1(ku322);ced-3(n717);SCM::gfp* |
| *C06G1.5 (tm5279)* | | *ced-3(n717);SCM::gfp*  *Is[lin-28::gfp::lin-28 3’UTR;rol-6]*  *ced-3(n1286);Is[lin-28::gfp::lin-28 3’UTR;rol-6]*  *Is[lin-28(D31A)::gfp::lin-28 3’UTR;rpl-28::RFP;rol-6]* |
| *Y95B8A.6(tm5312)* | | **miR/*ced-3* Enhancer Screen** |
| *sod-3(tm760)* | | *lsy-6(ot71)* |
| *bath-43 (tm770)* | | *mir-1(n4101)* |
| *rap-1 (pk2082)* | | *mir-1(n4102)* |
| *Y67D8C.5 (ok1575)* | | *mir-124(n4255)* |
| *glh-1(gk100)* | | *mir-2(n4108)* |
| *vap-1(ok392)* | | *mir-228(n4382)* |
| *F16B12.6 (gk1118)* | | *mir-230(n4535)* |
| *chk-2(gk212)* | | *mir-231(n4571)* |
|  | | *mir-232(nDf56)* |
|  | | *mir-233(n4761)* |
| **miR/*ced-3* Enhancer Screen** | **miR/*ced-3* Enhancer Screen** |  |
| *mir-234(n4520)* | *mir-47(gk167)* |  |
| *mir-235(n4504)* | *miR-48(n4097)* |  |
| *mir-237(n4296)* | *mir-48; mir-241(nDf51)* |  |
| *mir-238(n4112)* | *mir-51(n4473)* |  |
| *mir-239a-b (nDf62)* | *mir-52(n4100)* |  |
| *mir-240&mir-786(n4541)* | *mir-52(n4114)* |  |
| *mir-241(n4316)* | *mir-53(n4113)* |  |
| *mir-242(n4605)* | *mir-54&mir-55&mir-56(nDf58)* |  |
| *mir-243(n4759)* | *mir-58(n4640)* |  |
| *mir-244(n4367)* | *mir-59(n4604)* |  |
| *mir-245(n4798)* | *mir-60(n4947)* |  |
| *mir-246(n4636)* | *mir-61; mir-250 (nDf59)* |  |
| *mir-247&mir-797(n4505)* | *mir-62(n4539)* |  |
| *mir-249(n4983)* | *mir-63(n4568)* |  |
| *mir-251(n4606)* | *mir-64, mir-229 (nDf52)* |  |
| *mir-252(n4570)* | *mir-64_66,mir-229(nDf63)* |  |
| *mir-253(nDf64)* | *mir-67(n4899)* |  |
| *mir-254(n4470)* | *mir-70(n4109)* |  |
| *mir-256(n4471)* | *mir-70(n4110)* |  |
| *mir-257(n4548)* | *mir-71(n4115)* |  |
| *mir-258(n4797)* | *mir-72(n4130)* |  |
| *mir-259(n4106)* | *mir73-74(dDf47)* |  |
| *mir-260(n4601)* | *mir-75 mir-79(n4472, n4126)* |  |
| *mir-261(n4594)* | *mir-75(n4472)* |  |
| *mir-265(n4534)* | *mir-76(n4474)* |  |
| *mir-268(n4639)* | *mir-77(n4286)* |  |
| *mir-269(n4641)* | *mir-78(n4637)* |  |
| *mir-270(n4595)* | *mir-79(n4126)* |  |
| *mir-273(n4438)* | *mir-80,mir-227(nDf53)* |  |
| *mir-34(n4276)* | *mir-81&mir-82(nDf54)* |  |
| *mir-35-41 (nDf50)* | *mir-83(n4638)* |  |
| *mir-355(n4618)* | *mir-84 (n4037); lin-58(n4097)* |  |
| *mir-357-8(nDf60)* | *mir-84(n4037)* |  |
| *mir-359(n4540)* | *mir-85(n4117)* |  |
| *mir-360(n4635)* | *mir-86(n4607)* |  |
| *mir-42-44 (nDf49)* | *mir-87(n4104)* |  |
| *mir-45(n4280)* | *mir-87, mir-233(n4104, n4761)* |  |
| *mir-46(n4475)* |  |  |
| *mir-46-47(n4475;gk167)* |  |  |
